# Soil Mycobiome Is Shaped by Vegetation and Microhabitats: A Regional-Scale Study in Southeastern Brazil

**DOI:** 10.3390/jof7080587

**Published:** 2021-07-22

**Authors:** Danielle Hamae Yamauchi, Hans Garcia Garces, Marcus de Melo Teixeira, Gabriel Fellipe Barros Rodrigues, Leila Sabrina Ullmann, Adalberto Garcia Garces, Flavia Hebeler-Barbosa, Eduardo Bagagli

**Affiliations:** 1 Department of Chemical and Biological Sciences, Institute of Biosciences, São Paulo State University ‘Júlio de Mesquita Filho’, Botucatu 18618-689, SP, Brazil; atiweb@gmail.com (H.G.G.); atiestorage@gmail.com (A.G.G.); 2 Center for Tropical Medicine, Faculty of Medicine, University of Brasília (UnB), Brasília 70910-900, DF, Brazil; marcus.teixeira@gmail.com; 3 Department of Biostatistics, Plant Biology, Parasitology and Zoology, Institute of Biosciences, São Paulo State University ‘Júlio de Mesquita Filho’, Botucatu 18618-689, SP, Brazil; gfellipe5@gmail.com; 4 Institute for Biotechnology, São Paulo State University ‘Júlio de Mesquita Filho’, Botucatu 18607-440, SP, Brazil; leila_ullmann@yahoo.com.br; 5 Laboratory of Molecular Biology, Medical School, São Paulo State University ‘Júlio de Mesquita Filho’, Botucatu 18618-687, SP, Brazil; flavia.hb.trovao@unesp.br

**Keywords:** soil mycobiome, NDVI, fungal communities

## Abstract

Soil is the principal habitat and reservoir of fungi that act on ecological processes vital for life on Earth. Understanding soil fungal community structures and the patterns of species distribution is crucial, considering climatic change and the increasing anthropic impacts affecting nature. We evaluated the soil fungal diversity in southeastern Brazil, in a transitional region that harbors patches of distinct biomes and ecoregions. The samples originated from eight habitats, namely: semi-deciduous forest, Brazilian savanna, pasture, coffee and sugarcane plantation, abandoned buildings, owls’ and armadillos’ burrows. Forty-four soil samples collected in two periods were evaluated by metagenomic approaches, focusing on the high-throughput DNA sequencing of the ITS2 rDNA region in the Illumina platform. Normalized difference vegetation index (NDVI) was used for vegetation cover analysis. NDVI values showed a linear relationship with both diversity and richness, reinforcing the importance of a healthy vegetation for the establishment of a diverse and complex fungal community. The owls’ burrows presented a peculiar fungal composition, including high rates of Onygenales, commonly associated with keratinous animal wastes, and Trichosporonales, a group of basidiomycetous yeasts. Levels of organic matter and copper influenced all guild communities analyzed, supporting them as important drivers in shaping the fungal communities’ structures.

## 1. Introduction

The kingdom Fungi encompasses a legion of heterotrophic and eukaryotic organisms, considered essential for all life on Earth [1,2]. Besides acting as the primary decomposers of organic matter, fungi also play key roles as mutualists and pathogens, both of autotrophs and heterotrophs, including mammals [3,4]. Despite their recognized importance for ecology, agriculture, biotechnology, human and animal health, some basic and fundamental aspects of fungal biology remain incompletely elucidated. For instance, the global numbers of fungal species have been estimated to be from 1.5 to 5.1 million, though only around 150,000 have been adequately described [2,5,6,7,8]. 

The general patterns of fungal diversity on both large and local geographic scales are still poorly comprehended, since large-scale sampling mycobiome studies are scarce [7,9,10,11], most of them are regional and do not cover all biogeographic areas [12,13,14,15,16,17]. Nevertheless, increasing amounts of data indicate that fungi exhibit distinct and complex biogeographic patterns [14,15,18], contrasting with the “everything is everywhere” hypothesis considered for microbes with lengths less than 2 mm [19,20]. The Northern and Southern hemispheres present clear biogeographical effects on the fungal kingdom, as with plants and animals, according to Gondwanan origins [15]. Therefore, bioclimatic components such as mean annual temperature and precipitation, latitude effect and vegetation are key drivers of fungal diversity and distribution patterns on a global scale [14,17,21]. 

Besides the large-scale biogeographic effects, the local- and regional-scale heterogeneity of soil microhabitats has been identified as one of the most important drivers of diversity for soil-dwelling microbes [20,22]. The soil, or pedosphere, is one of the most complex and dynamic systems that balances long-term stability with continuous microhabitat perturbation, in a near ideal condition for microbial life diversification [3,23]. Deciphering the key ecological and evolutionary forces that create and maintain biological diversity is pivotal, though equally urgent is understanding the processes that erode and induce losses of diversity, especially those associated with human action in nature in the recent times of the Anthropocene Epoch [24,25]. Deforestation, agricultural activities, soil erosion, pollution and associated factors impact negatively on biodiversity [26,27,28], but the exact extensions and underlying processes involved are not entirely dimensioned, precluding scientifically based actions that might mitigate the problems [29,30]. Furthermore, the connections between wild animals and soil biodiversity are poorly elucidated. Some studies in Australia demonstrated that terrestrial vertebrates promote diversity of arbuscular mycorrhizal fungi in a rainforest soil located in north-eastern Queensland [31]. The reintroduction of locally extinct species contributes to ecosystem restoration by altering soil fungal communities in degraded areas [32]; and those wild digging mammals (marsupials) contribute positively to rhizosphere fungal community composition and seedling growth [33].

The soil fungal diversity in southeastern Brazil (South America), that is located in a transitional region that harbors patches of distinct biomes and ecoregions, under the Neotropical realm, has been under-measured [25,34]. This regional-scale study includes samples from different vegetation cover, such as: subtropical moist broadleaf forest (a semi-deciduous forest), savanna and pasture fields, coffee and sugarcane plantations, abandoned rural buildings, and also from animal burrows, such as those constructed by the burrowing owl *Athene cunicularia* (Strigiformes, Strigidae) and the nine-banded armadillo *Dasypus novemcinctus* (Cingulata, Dasypodidae). The burrowing owl is a bird of prey, nesting exclusively underground, that occurs in both South and North America, including urbanized areas [35]. Besides small rodents, the owls also catch and eat insects, which are attracted to dung that the birds collect and deposit at the burrows’ entrance [36]. The nine-banded armadillo is a peculiar medium-sized mammal, remnant of one the most exclusive and once dominant animal groups (Xenarthra) in South America, since the Eocene epoch, around 50 mya [37,38]. Besides intense foraging activities in the upper-soil horizons (O and H) to collect and eat worms, insects, termites and small animals, the nine-banded armadillos present strong digging abilities and construct great numbers of burrows, channels and under-soil small caves for protection [39]. There are scarce studies related to burrowing wild animal influence on soil fungal communities [40], and none in our region. Herein, we aim to evaluate the soil fungal diversity in the previously described region and to determine the influence of the vegetation, edaphic factors and the presence of burrowing wild animals over the patterns of local fungal diversity. 

## 2. Materials and Methods

### 2.1. Study Area and Sampling 

**Figure 1:**
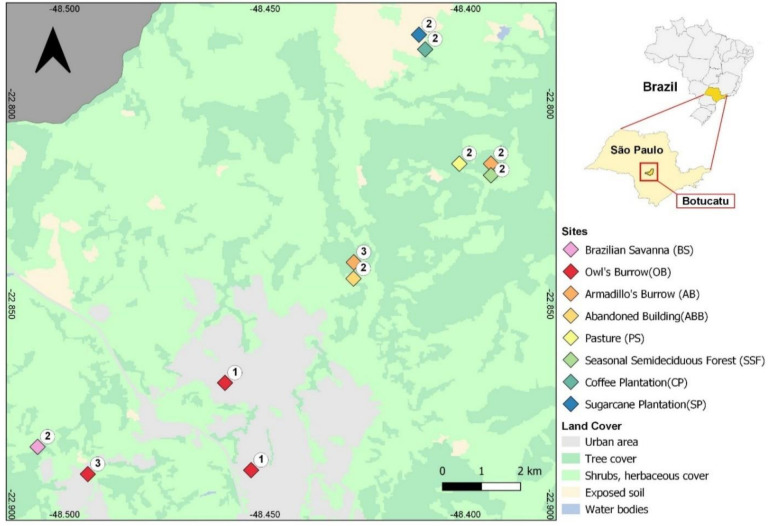
Map of study area and sample points, Botucatu, São Paulo, Brazil. The map was generated with the software QGIS version 3.6 Noosa (QGIS Development Team, 2019. Geographic Information System of QGIS. Geospatial Foundation Open Source Project). The number of collected samples for one season are notated at each collection point (22 samples).

The focused 109 km^2^ study area (Figure 1) is located in southeastern Brazil, under the Neotropical realm, in a transitory zone that harbors distinct ecoregions influenced by two current biomes (tropical and subtropical dry broadleaf forests, and tropical and subtropical grasslands, savannas and shrublands), as well as by the historical biome (temperate broadleaf and mixed forests) then existing during the Last Glacial Maximum in the Quaternary period [25,34]. The altitude is about 830 m above sea level, with a mean annual temperature and precipitation of 19.1 °C and 1324 mm, respectively. It contains natural vegetation, agricultural, pasture and reforestation fields, and an urban area (Botucatu, São Paulo state, Brazil—with 146,000 inhabitants). The soil samples include materials from armadillos’ burrows (AB), owls’ burrows (OB), seasonal semi-deciduous forest (SSF), Brazilian savanna or Cerrado (BS), pasture (PS), coffee plantation (CP), sugarcane plantation (SP) and abandoned rural buildings (ABB). All the samples from the armadillos’ burrows were collected in two areas of semi-deciduous forest, where these animals use to live. On the other hand, owls live in pasture or urban areas. The owl’s burrows samples were obtained from urban areas with scarce vegetation (grass). 

The samples were collected at two distinct moments, when weather conditions had been relatively stable, with no rain for at least three weeks, during the autumn (2 and 3 May 2018) and spring (30 and 31 October 2018) in the Southern Hemisphere. The total of 44 samples evaluated included 22 from the first collection (autumn) and 22 from the second collection (spring) (Table S1). For SSF, BS, PS, CP, SP and ABB, four samples were collected, two samples in each period (autumn and spring). For burrowing animals (AB and OB), five samples were collected in each period. As the AB and OB have never been studied in our region, an additional sampling was made in order to establish a better characterization of fungal communities associated to these microhabitats. 

A metal shovel was utilized for the soil collections, and was carefully cleaned with cotton soaked in 70% alcohol before each sampling. The burrow samples were collected at a distance of up to 50 cm from the burrow entrance. The other samples were collected at a depth of 0–10 cm, after removing the superficial layers of litter, vegetable fragments or grass (in pastures). The individual samples for DNA extractions were placed in individual sterile collection flasks with lids (volume of 80 mL) and maintained protected in an isothermal box. Composite soil samples (400 g, from each collection site) were also prepared by mixing 3 distinct soil aliquots obtained (3–5 m distant from each other) at horizon H (0–10 cm depth), or from 2–3 distinct burrows (0.5 m depth, for armadillo and owl samples), conditioned in appropriate bags and sent to the Laboratory for Soil Analysis at the Agricultural Sciences Faculty (FCA/UNESP), to perform the physico-chemical analysis (Table S1). The mixed soil aliquots were obtained from the same geographic area as shown in Figure 1.

The coordinates for each collection point were obtained using a GPS 12 Personal Navigator^®^ (Garmin^®^, Schaffhausen, Switzerland). The collection points are located on the map (Figure 1). The map was generated in the software QGIS version 3.6 Noosa (QGIS Development Team, 2019. Geographic Information System of QGIS. Geospatial Foundation Open Source Project) (http://qgis.osgeo.org, accessed on 20 July 2021).

Normalized difference vegetation index (NDVI) was used for vegetation cover analysis. This index measures primary productivity, quantitatively, allowing an estimation of vegetation health [41]. NDVI is often used to monitor seasonal and temporal vegetation change and enables spatial comparisons [41,42,43,44].

Image for NDVI analysis was acquired by a Sentinel-2 MultiSpectral Instrument (MSI), provided by the European Space Agency (ESA). These images were obtained close to collection date (10 May and 22 October 2018), on the tile T22KGV, under cloud-free conditions (cloud cover less than 20%). Atmospheric correction was performed using the Semi-Automatic Classification Plugin Documentation plugin [45], available in QGIS version 3.6 Noosa software. NDVI was calculated using near-infrared spectral bands (NIR, band 8) and red spectral bands (RED, band 4), being computed by: NDVI = (NIR − RED)/(NIR + RED) [41].

### 2.2. DNA Extraction and Sample Processing 

The DNA was extracted using the MoBio DNeasy PowerSoil Kit (PowerSoil^®^DNA Isolation Kit), following the manufacturer’s instructions. The vortex was replaced by Precellys^®^ 24 (Bertin Instruments, 5 cycles of 3 × 40 s). The first PCR stage utilized the primers 5.8-Fun (5′- AAC TTT YRR CAA YGG ATC WCT-3′) and ITS4-Fun (5′- AGG CCT CCG CTT ATT GAT ATG CTT AAR T-3′), which amplify the internal transcribed spacer 2 (ITS2) region of the nuclear ribosomal DNA [46]. The primers were previously modified by inserting the Illumina adapter sequence at the 5 ‘end, allowing the indexing in the second PCR reaction [47]. These primers were designed to optimize the amplification of Eumycotas for working on several studies, proving to be efficient to amplify several fungi and also restricted concerning the amplification of other eukaryotic organisms [46].

For each sample, 5 µL of DNA (5 ng/µL) was amplified using KAPA Taq HotStart PCR Kit (Roche, Basiléia, Switzerland), at final reaction volume of 25µL. The thermal cycler (Veriti 96-Well Thermal Cycler, Applied Biosystems^®^, Waltham, MA, USA) was adjusted to: 95 °C for 3 min, followed by 35 cycles at 98 °C for 20 s, 55 °C for 20 s, 72 °C for 30 s and a final extension at 72 °C for 1 min. The amplification was verified in electrophoresis gel and purified using Beckman Coulter AMPure XP beads (Danaher Corporation, Washington, DC, USA). 

Indexing was performed using the Nextera XT Index Kit (96 indexes, 384 samples) (Illumina^®^, San Diego, CA, USA). The cycle used was 95 °C for 3 min, 8 cycles at 95 °C for 30 s, 55 °C for 30 s, 72 °C for 30 s and 72 °C for 5 min for a final extension. The purified product was quantified using Qubit ™ (Invitrogen, Waltham, MA, USA). The size of the library was measured using a Bioanalyzer (Agilent Technologies, Santa Clara, CA, USA) and the concentration of each sample was readjusted to 4 nM manually. For sequencing, a MiSeq Reagent Kit v3 (600-cycle) Illumina was used at a final concentration of 15 pM and 25% PhiX Library. The obtained raw fastq data was deposited in the Sequence Read Archive (SRA) repository under accession number PRJNA679063.

### 2.3. Data Processing

The retrieved fastq files were processed using the AMPtk pipeline [48]. Initially, the primer sequences, adapters and low-quality nucleotide bases were purged and used for the paired-end joining step. This software processes the data using USEARCH [49] and VSEARCH [50]. Clustering and denoising were done through UPARSE and UNOISE3, respectively. The singletons were discarded to minimize errors. Taxonomy classification in OTU (Operational Taxonomic Unit) was made by using the UNITE 8.0 database (https://doi.org/10.15156/BIO/786349, accessed on 20 July 2021) employing 97% similarity for classification (Table S2). For the classification of taxa into guilds, we used FUNguild [51]. After classification, the guilds were separated into four main groups: saprotrophs, animal pathogens, plant pathogens and plant symbionts.

### 2.4. Statistical Analysis

To verify whether the sample size was sufficient, rarefaction curves were plotted using the iNext function for each of the fungal communities studied. The alpha diversity was estimated using OTU richness (S) and the Shannon index (H′). The richness (S) and Shannon values were analyzed for normality (Shapiro–Wilk test) and homoscedasticity (Levene test). Kruskall–Wallis was utilized for comparison of among sample types (*p* < 0.05) with the a posteriori Dunn test, while ANOVA was utilized for comparison of diversity for each different sample type (*p* < 0.05) with the a posteriori Tukey test. Dissimilarity in the community structure was accessed by constructing a similarity matrix, which compared the locations, using the Bray–Curtis dissimilarity coefficient. This dissimilarity was plotted on a non-metric multidimensional scale (nMDS), which places sample points in a two-dimensional space. A cluster analysis was employed to describe the environments, based on abundance of taxa between samples (beta diversity). A permutational multivariate analysis of variance (PERMANOVA) [52] was utilized to compare the fungal communities between different environments and guilds with the *a posteriori* test for pairwise comparison between the sampled environments (Table 1). To verify the species–environment relationship and correlations with environmental variables, we performed a canonical correspondence analysis (CCA) [53]. Forward and backward selection was performed with the ordistep R function to determine the best fitting model (lowest AIC) for explaining the dispersion of samples within the fungal guilds. Environmental variables that were identified from “ordistep” were used as explanatory variables in CCA plots. The statistical significance of eigenvalues of the CCA axis was evaluated by randomization (Monte Carlo) tests, using 9999 runs for each analysis. The relationship between NDVI (explanatory variable) and fungal richness was tested through a linear mixed model (lmm). The same test was applied for observing the relation between NDVI and diversity (H′). For both tests, the habitat and season were treated as a random effect to control the geographic singularities of fungal communities. All analyses were performed using the statistical program R (R Core Team, 2019), with the “stats” packages for univariate analysis (R Core Team, 2019), “vegan” for multivariate analysis, “lme4” for linear mixed models and diversity measurement [54] and “iNEXT” for rarefaction [55].

## 3. Results

The soil samples included materials from armadillos’ burrows (AB), owls’ burrows (OB), seasonal semi-deciduous forest (SSF), Brazilian savanna or Cerrado (BS), pasture (PS), coffee plantation (CP), sugarcane plantation (SP) and abandoned rural buildings (ABB). Normalized difference vegetation index (NDVI) values around one, which correspond to healthy vegetation, were found in forested areas like AB and SSF samples. Low NDVI values (near zero) were found in OB and ABB samples, showing a sparsely vegetated area associated to anthropized environment.

**Figure 2:**
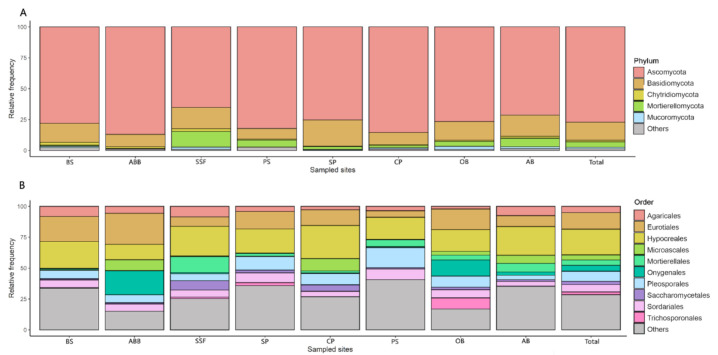
Relative frequency of phylum (**A**) and the ten most abundant orders (**B**) in different environments. BS = Brazilian savanna; ABB = abandoned rural building; SSF = seasonal semi-deciduous forest; PS = pasture; SP = sugarcane plantation; CF = coffee plantation; OB = owls’ burrows; AB = armadillos’ burrows.

A total of 11,060,447 reads were obtained containing 8154 OTUs, with a median of 1156 OTUs ± 448.57 SD per sample after data-processing including a quality filter. Sampling sufficiency was reached for most of the environments, achieving or coming close to the extrapolated richness estimated at 2.5 million reads, as shown by the rarefaction curve (Table S4/Figure S1). Most of the OTUs found (99.73%) belonged to the Fungi kingdom. The phylum with the highest frequency was Ascomycota (77.15%), followed by Basidiomycota (14.62%). Found in a smaller amount were some representatives of the old Zygomycota (Mortierellomycota with 4.35% and Mucoromycota with 1.26%), Chytridiomycota (1.30%) and others (1.32%, including Glomeromycota) (Figure 2A). Mortierellomycota showed a significant increase in its relative abundance in soils from SSF (12.73%) and AB (6.9%). Some orders, such as Hypocreales (20.83%), Eurotiales (13.41%) and Pleosporales (8.2%) were the most dominant. Sordariales, Agaricales, Onygenales, Mortierellales, Microascales and Saccharomycetales also showed high frequencies (Figure 2B). These ten most frequent orders constituted almost 70% of the total reads, although there is a variation in the relative frequency of orders depending on the environment.

**Figure 3:**
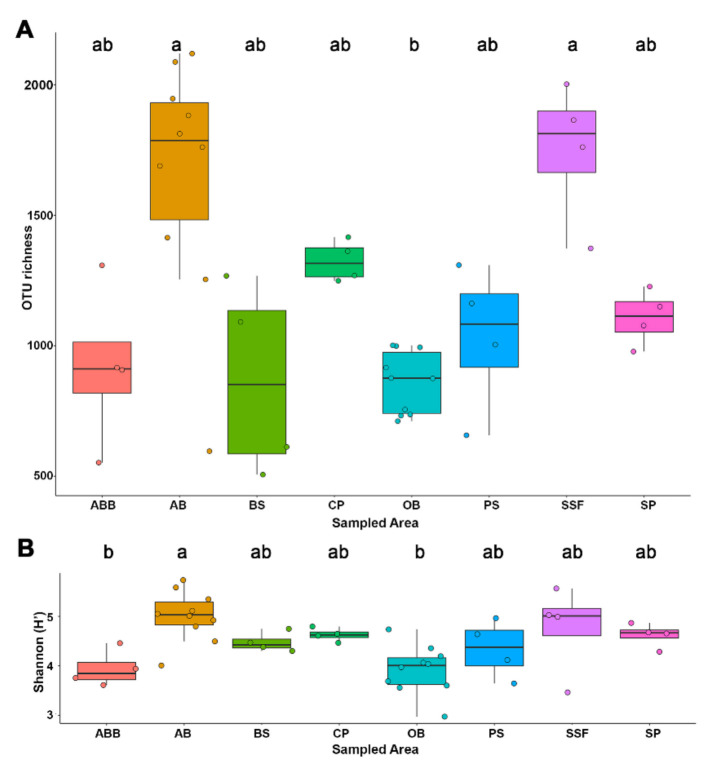
Boxplot showing richness (**A**) and Shannon index diversity for each habitat (**B**). ABB = abandoned rural building; AB = armadillos’ burrows; BS = Brazilian savanna; CF = coffee plantation; OB = owls’ burrows; PS = pasture; SSF = seasonal semi-deciduous forest; SP = sugarcane plantation. Different letters are significantly different at Dunn (richness) and Tukey tests (Shannon index), *p* < 0.05.

Both, fungal richness (β = 2.77, *t =* 5.29, *p* < 0.001) and diversity (H’) (β = 2.53, *t =* 4.92, *p* < 0.001), were positively associated with NVDI (Figure S2). Higher richness values were observed in environments with healthy vegetation (highest NDVI values) such as AB and SSF (mean 1656 OTUs ± 461.71 and 1750 OTUs ± 270.48, respectively). Sparsely vegetated areas, such as OB (859 OTUs ± 118.32) and ABB samples (921 OTUs ± 308.84), showed the lowest richness, together with BS (869 OTUs ± 368.01). The richness of OB samples showed significant difference compared with AB and SFF samples (*p* < 0.05). It was observed that environments with high richness (Figure 3A) presented a high alpha diversity as well (Shannon index—Figure 3B). ABB and OB samples, considered the most anthropized environments, had the lowest alpha diversity indexes (3.94 ± 0.37 and 3.92 ± 0.48, respectively), showing a significant difference compared with the AB sample (5.01 ± 0.50) (*p* < 0.05). Other environments like SP, CP and PS showed intermediate values of richness (1108 ± 106.12; 1315 ± 91.15; 1033 ± 279.90, respectively) and diversity (4.62 ± 0.24; 4.63 ± 0.13; 4.34 ± 0.38) (Figure 3). Both the diversity and richness showed a linear relationship with NDVI values, independent of local and temporal factors.

**Figure 4:**
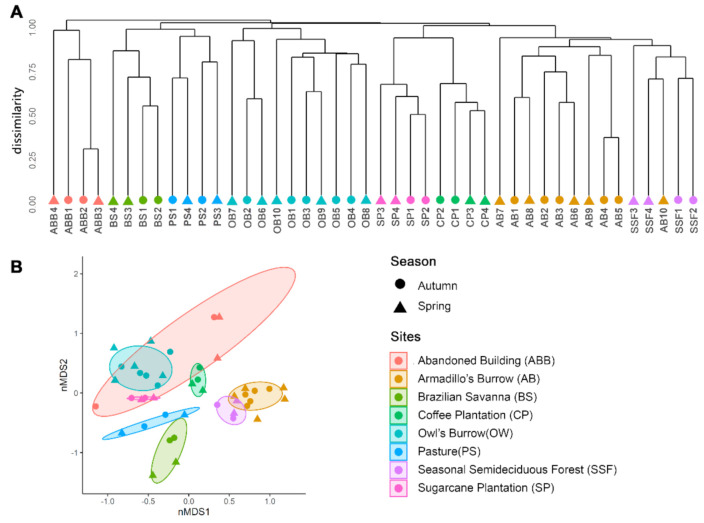
Cluster analysis (**A**) and non-metric multidimensional scale analysis (nMDS) (**B**) using the Bray–Curtis dissimilarity coefficient, with samples grouped by environment, showing the similarity of the fungal community structure according to habitat for both seasons (autumn represented by a circle and spring represented by a triangle).

The cluster analysis (Bray–Curtis dissimilarity) grouped the samples by environment, except in one AB sample (AB10), showing the similarity of the fungal community structure according to environmental variation (Figure 4A). Furthermore, it was not possible to differentiate the groups according to the collection periods (autumn- and spring- seasonal variation). The PERMANOVA test was applied both in fungal communities and in individual guilds (Table 1/Table S3). The owls’ and armadillos’ burrows were the most distinctive communities, differing significantly in beta diversity. Significant differences were observed between OB–AB, PS–AB and CP–OB (Table 1, *p*-adjusted < 0.05). The similarity or differences of the mycobiome were graphically represented in nMDS. The ellipse overlaps in two axes of sorting space indicated similarity within a given environment (Figure 4B).

The soil chemical analysis is displayed in Table S1. Lower pH values can be observed in BS (pH 4.25) and ABB (pH 4.3) related to other environments (pH 5.25–6.35). In addition, BS showed a high value of aluminum cation (Al^3+^) (5.25 mmolc/dm^3^). Organic matter (OM) reaches the highest value in SSF samples and the lowest in OB and BS samples. The variation of copper (Cu) values was within 0.525–5.85 mg/dm^3^, showing lower values in BS, OB and SP. Manganese (Mn) and magnesium (Mg) showed high concentrations in ABB, AB and SST.

**Figure 5:**
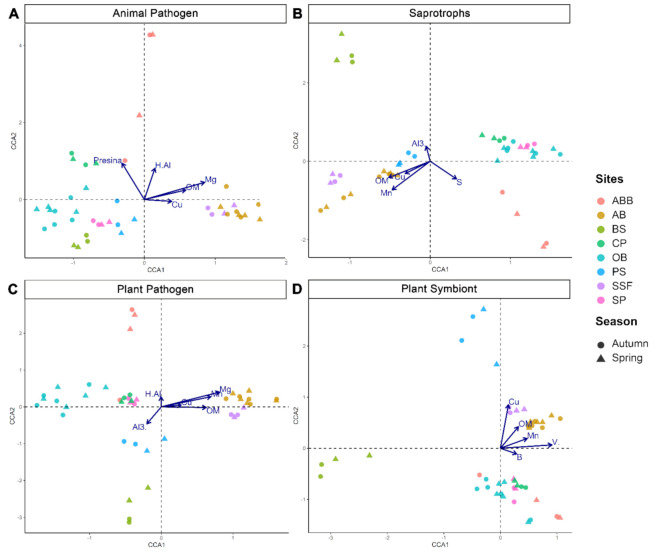
Canonical correspondence analysis (CCA) for guilds to verify the species–environment relationship separated by sample sites and correlations with environmental variables. Autumn = circle; spring = triangle; AB = armadillos’ burrows; BS = Brazilian savanna; CF = coffee plantation; OB = owls’ burrows; PS = pasture; SSF = seasonal semi-deciduous forest; SP = sugarcane plantation; OM = organic matter; Cu = copper; Mg = magnesium; Presina = resin phosphorus test; H.Al = potential acidity (H + Al); Al3. = aluminum cation; Mn = manganese; S = sulfur; V. = base saturation; B = boron. Guilds: (**A**) Animal Pathogen; (**B**) Saprotrophs; (**C**) Plant Pathogen; (**D**) Plant Symbiont.

The canonical correspondence analysis (CCA) showed the environmental factors that influenced the guild community. Organic matter (OM) and copper (Cu) influenced the community of all four guilds analyzed, positively correlating with SSF and AB (Figure 5). Manganese (Mn) showed a positive correlation with SSF and AB in saprotroph, plant pathogen and plant symbiont guilds. Furthermore, magnesium (Mg) positively influenced SSF and AB in animal pathogen and plant pathogen guild communities. BS was positively correlated with aluminum cations (Al^3+^) in saprotroph and plant pathogen guilds. 

## 4. Discussion

In this study we provided baseline data on soil fungal diversity in a poorly studied region in southeastern Brazil that congregates distinct ecological conditions in terms of vegetation, soil type, land use and the presence of burrowing wild animals. Our data showed a complex fungal diversity pattern, with most fungi being classified as Ascomycota and Basidiomycota. In fact, these two terrestrial fungal groups have been observed as dominants in other similar mycobiome studies [9,12,14,17,21,40,56]. Our data also revealed a higher relative abundance of Ascomycota (ranging from 65.24 to 87.03%) when compared with Basidiomycota (Figure 2A). The predominance of Ascomycota in the studied region must be reflecting a pattern of the global distribution of fungal biodiversity already proposed by Tedersoo et al. [14], which observed a peak in the richness of saprotrophic Ascomycota in tropical ecosystems. In addition, ectomycorrhizal fungi, often related to Basidiomycota, have their peak of richness in temperate forests and Mediterranean biomes, in the middle latitudes of the Northern Hemisphere (40° to 60° N) [14]. 

Saprotrophic fungi were dominant in our data, including the fungal orders Hypocreales, Sordariales, Microascales (class Sordariomycete), Eurotiales, Onygenales, Chaetothyriales (class Eurotiomycetes) and Pleosporales (class Dothideomycetes) (Figure 2B), considered prevalent in tropical ecosystems [14]. The saprotrophs are well adapted to long-term survival and grow in a broad spectrum of substrates and bioclimatic factors. By possessing a wide repertoire of enzymes to process organic compounds, and producing high numbers of spores, saprotrophic fungi tend to occur as dominant groups in diverse environments [14,21,56]. However, it has been suggested that the structure of fungal communities is not uniform, but rather presents significant variations of the dominant groups in the different communities, in contrast to bacterial communities that tend to be more uniform [57]. 

Basal fungi, formerly known as Chytrids and Zygomycota, were observed at low rates in most samples, except Mortierellomycota, which was moderately detected in the environments that presented the highest fungal richness: the seasonal semi-deciduous forest (SSF) and armadillos’ burrows (AB). The low detection of Glomeromycota in our samples might also be associated with some limitation of the primers used in detecting the peculiar intrasporal rDNA polymorphism in this fungal group [58]. The richness of Mortierellomycota species often peaks under milder environmental conditions, characterized by a greater availability of organic nutrients, higher moisture contents and colder temperatures [14,21,59]. A high proportion of Mortirellomycota in SST and AB might be related to the afforestation in these areas. The vegetation has a protective effect, bringing a decrease in temperatures, and increases in both organic debris and humidity [21,59,60]. This is an additional example of how the structure of the fungal community might reflect the environmental factors or vice versa. 

The lowest fungal richness occurred in soil samples from owls’ burrow(OB), collected in urban areas with sparse vegetation (grass) and showing the lowest organic matter (OM) content. Soils with very low carbon levels could let to reductions in both microbial biomass and diversity [10]. Additionally, abandoned rural buildings (ABB) and Brazilian savanna (BS) showed low richness. Although the abandoned buildings’ soil samples presented organic materials, represented by rodent feces, insects and remainders of animal food stock, the local humidity was low and the vegetation was very rare or completely absent. The sandy soil prevalent in Brazilian savanna has less capacity to retain water, and is associated with low values of organic matter, a condition that might favor generalist saprophagous fungi. In addition, the lowest pH values were observed in both of these environments (BS and ABB) (Table S1), and certainly influence directly or indirectly the prevalence of some fungi. Most fungi tolerate pH variation [61,62], but this can alter the nutrient availability, vegetation growth or local microbiota (as a prevalence of bacteria), causing changes in the fungal community [57,63,64]. Related to fungal diversity, it was observed that higher Shannon indexes were present in soils of the Brazilian savanna than those of abandoned buildings and owls’ burrows. This higher fungal diversity in BS samples might be explained the presence of non-dominant communities. The Brazilian savanna is characterized by a strong seasonality pattern (drought and rain), with occasional fires in dry seasons. The typical cerrado vegetation of the Brazilian savanna is quite diverse and adapted to such conditions, as well as to soil-related factors, such as low pH, low organic matter and aluminum toxicity [65,66]. Thus, temporal heterogeneity might be an important driver for more diverse communities found in BS, since this does not enable the dominance of a few groups (each species is favored in a different period) [67]. Furthermore, the diversity of trees, shrubs and herbaceous plants in Brazilian savanna results in a relatively heterogeneous environment that might contribute to a relative increase in the fungal diversity. 

NDVI values were in accordance with the observed environment vegetation. High NDVI values were observed in soils from SSF and AB, both located in forested areas, while low NDVI values were found in soil samples of OB and ABB, both with sparse vegetation and bare soils. Vegetation cover had a positive correlation with richness and diversity. This reinforced the importance of a healthy vegetation and a high primary productivity for establishing a complex and diverse mycobiome [11]. On the other hand, soil samples from sugarcane and coffee plantations (agricultural areas) also presented relatively high Shannon values. Heterogeneous environment and low anthropogenic impact are factors that might contribute to increasing local diversity in forests [59,67,68,69]. These heterogeneous environments can provide differentiated organic matter and a high percentage of plant litter, which favor the establishment of decomposing fungi. These factors, coupled with the high root density characteristic of mature forests, contribute to the establishment of symbiotic fungi, such as mycorrhizal fungi [59,60]. Furthermore, forests can be strongly associated with high local diversity, justified by high humidity and the protection that local vegetation offers against sunlight (UV) and sudden variations in temperatures [14,21,70]. It is not surprising that the highest richness and diversity were found in the AB and SST samples, since they were collected from areas with these conditions. In agricultural areas, the presence of vegetation might exert strong influences through root exudates, plant litter and variation of soil moisture [71]. These cultivation areas allow the establishment of vegetation-associated fungi (i.e., mycorrhizal fungi and endophytes), increasing the richness and diversity of these environments [71,72]. Additionally, Bastida et al. [10] suggested that moderate reductions in soil carbon content of high carbon soils might reduce the microbial biomass increasing microbial diversity. This reduction of soil carbon may be observed in deforested areas and crops. Thus, cultivated areas that have cover vegetation, like crops, might increase the fungal diversity when compared to less vegetated areas.

The CCA analysis revealed the influence of several environmental factors that have an impact on the guild community (Figure 5). Organic matter and copper (Cu) influenced the community of all guilds. Copper, widely used in industry and agriculture, has been shown to be an influential factor in the microbial community in several studies, often showing negative effects on the diversity of microorganisms and impact on their activities [72,73,74,75]. Thus, the Cu concentration might be an influential factor in fungal communities herein analyzed, showing a positive correlation with SST and AB samples in all guilds. The influence of organic matter is also described as a factor that increases the abundance of decomposing microorganisms [76]. In addition to the quantitative difference, this might represent a qualitative divergence that could result in the establishment of specialized fungi [59,77,78].

By using a cluster analysis based on Bray–Curtis, a similarity was found among soils collected from the same environment, grouping almost all the samples by environment (Figure 4). The geographic location of these places can explain the grouping of the SSF samples with the AB samples since the armadillos’ burrows were inserted within the forest, presenting similar environmental conditions. Interestingly, AB was grouped separately from SSF with the exception of one sample, despite being collected in the same forest fragment. This is a shred of evidence that fungal communities proceeding from the same environmental conditions should have similar community structure, but the micro-habitat also can influence this structure. 

The collection periods (autumn- and spring-seasonal variation) showed no influence on fungal community, pointing out that the changes of these communities might be more strongly associated with the collection environment than the collection date. Vargas-Gastélum et al. [40] observed a difference between fungal communities collected in different seasons (winter and summer), especially in topsoil samples. Nevertheless, we must consider that our samples were collected in two periods only (May and November) during the transition of seasons and with the same relative climate in the two periods (autumn and spring season). Additional sampling would be necessary to enable robust statistical inferences. 

This is the first attempt at describing the soil fungal communities associated with habitats of owls and armadillos. The burrowing owl (*Athene curnicularia*) is a bird with a broad alimentation spectrum that ranges from insects to small vertebrates [79]. In most parts of the Americas, burrowing owls live in holes excavated into the ground within deforested locations, including urban areas [35,80], under intense human impact. The presence of the animal and its habits can change the microhabitat where it is inserted, resulting in a unique local fungal community [33,40,81]. The owl soil samples, whose relative frequencies of fungal orders differed in relation to other environments, presented a decreased frequency of Agaricales and substantial increases in species of Onygenales and Trichosporonales orders. The decreased frequency of Agaricales might be explained by the lesser abundance of arboreal vegetation surrounding the owl burrows where the soils were collected, which contain mainly grasses, shrubs and herbaceous vegetation [14,82,83]. The high frequency of Onygenales species reported herein is likely associated with the abundant presence of keratin-rich materials, probably generated by the own bird’s feathers and remains of small prey (including rodents) the owl captures; keratin is an abundant animal protein, whose degradation is carried out only by a few specialized groups, such as the Onygenales fungi [77,84,85]. The marked occurrence of Trichosporonales in owl soil samples is also noteworthy, since this basidiomycetous order of yeasts has been recognized as a general saprotropic associated with litter decomposition [86,87], and also contains an emerging group of species causing opportunistic human fungal infections [88,89]. 

The PERMANOVA test showed significant differences (*p*-adjusted < 0.05) between the fungal communities of OB and CP, of AB and PS and between the OB and AB. Furthermore, adjusted *p* values were very close to statistical significance in several other samples, when compared to burrow samples (Table S3). Similarly, PERMANOVA analyses from guild communities also differed significantly when compared to burrow samples (Table 1). As previously mentioned, the fungal community associated with owl and armadillo burrows may be the result of the differentiated microhabitat created in this type of environment, including parameters difficult to measure such as reduced exposure to the sun or variation in temperature, animal activity and differentiated organic material, like keratin or nitrogen-rich material, which may favor the establishment of some specialist fungi [33,40,81,84]. 

In addition, the PERMANOVA test found differences between AB and OB values in all performed analyses. Although both armadillos and owls construct burrows in soil, they are inserted into different environments. Armadillos live within or near forests, while the burrowing owl lives in open areas with moderate vegetation. These differences in the animals’ way of life (i.e., place where they construct the burrow) might determine the fungal community found in these samples. Therefore, the fungal communities in these habitats are strongly influenced by the surrounding environment into which they are inserted, while at the same time they are strongly influenced by animals’ activities. 

## 5. Conclusions

This work evidenced the influence of the environment on fungal communities even in nearby locations. Vegetation cover proved to be the key driver in establishing complex and diverse fungal soil communities. A sharp decrease in fungal diversity was observed especially when comparing forest samples with samples without or with sparse vegetation. Burrowing animals, birds and mammals, may be considered as additional drivers for soil fungal diversity. Knowledge of fungal diversity patterns can represent an important indicator of environmental changes. 

## Figures and Tables

**Table 1:** ANOVA, Kruskall–Wallis and PERMANOVA tests for diversity (H′), fungal richness (S) and fungal communities in different environments. A posteriori test for pairwise comparison between the sampled environments.

Pairwise		ANOVA * and Kruskall–Wallis *		PERMANOVA *				
Environment 1	Environment 2	H’	S	Fungal Communities	Animal Pathogen	Plant Pathogen	Plant Symbiont	Saprotroph
Armadillo’s Burrow	Coffee Plantation							
Armadillo’s Burrow	Abandoned Building	*			*		*	*
Armadillo’s Burrow	Brazilian Savana						*	*
Armadillo’s Burrow	Owl’s Burrow	*	*	*	*	*	*	*
Armadillo’s Burrow	Pasture			*		*		
Armadillo’s Burrow	Seasonal Semi-deciduous Forest					*		
Armadillo’s Burrow	Sugarcane Plantation					*		*
Owl’s Burrow	Coffee Plantation			*				*
Owl’s Burrow	Abandoned Building							
Owl’s Burrow	Brazilian Savana						*	
Owl’s Burrow	Pasture						*	
Owl’s Burrow	Seasonal Semi-deciduous Forest		*				*	
Owl’s Burrow	Sugarcane Plantation				*			*
Coffee Plantation	Abandoned Building							
Coffee Plantation	Brazilian Savana							
Coffee Plantation	Seasonal Semi-deciduous Forest							
Coffee Plantation	Sugarcane Plantation							
Coffee Plantation	Pasture							
Abandoned Building	Brazilian Savana							
Abandoned Building	Seasonal Semi-deciduous Forest							
Abandoned Building	Sugarcane Plantation							
Abandoned Building	Pasture							
Brazilian Savana	Seasonal Semi-deciduous Forest							
Brazilian Savana	Sugarcane Plantation							
Brazilian Savana	Pasture							
Seasonal Semideciduous Forest	Sugarcane Plantation							
Seasonal Semideciduous Forest	Pasture							
Sugarcane Plantation	Pasture							

* *p*.adjusted < 0.05.

## Data Availability

BioProject PRJNA679063: Fungal Metagenome from Brazilian soil.

## Supplementary Materials

The following are available online at https://www.mdpi.com/article/10.3390/jof7080587/s1, Figure S1: Rarefaction curve per habitat; Figure S2: Linear mixed model (lmm) showing the relationship between NDVI and fungal richness and relationship between NDVI and diversity; Table S1: Sampling data and soil physico-chemical analysis; Table S2: Rarefaction table; Table S3: PERMANOVA and *a posteriori* pairwise test; Table S4: Taxonomy classification of OTUs (Operational Taxonomic Unit) using UNITE database (97%).
