# Supplementary material for: Soil Mycobiome Is Shaped by Vegetation and Microhabitats: A Regional-Scale Study in Southeastern Brazil
Source: J Fungi (Basel). 2021 Jul 22;7(8):587. doi: 10.3390/jof7080587 (PMC8396882; doi:10.3390/jof7080587)

" Soil mycobiome is shaped by vegetation and microhabitats: a regional-scale study in southeastern Brazil "

Danielle Hamae Yamauchi, Hans Garcia Garces, Marcus de Melo Teixeira, Gabriel Fellipe Barros Rodrigues, Leila Sabrina Ullmann , Adalberto Garcia Garces, Flavia Hebeler-Barbosa, Eduardo Bagagli

FigureS1 Rarefaction curve per habitat extrapolated at 2.5 million reads. BS=Brazilian Savanna; ABB=Abandoned Rural Building; SSF=Seasonal Semideciduous Forest; SP=Sugarcane Plantation; CF=Coffee Plantation; PS=Pasture field; OB=Owl's Burrows; AB=Armadillo's Burrows

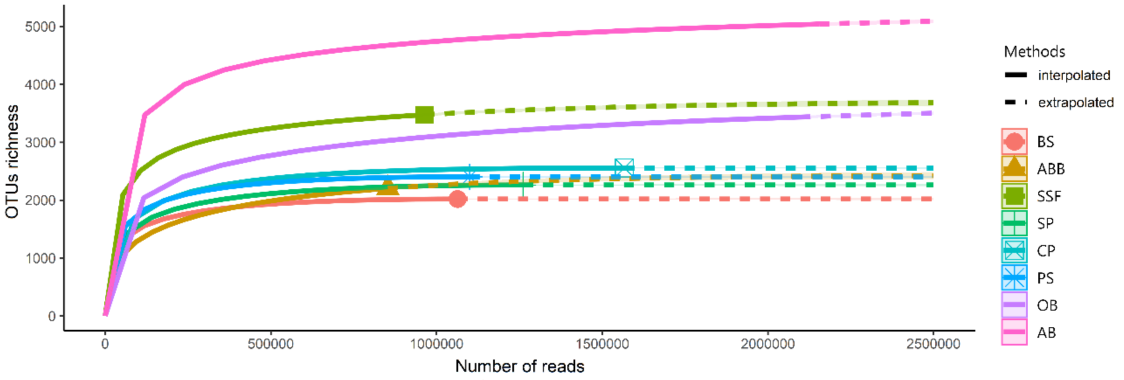

FigureS2 Linear mixed model (lmm) showing the relationship between NDVI (explanatory variable) and fungal richness (A) and relationship between NDVI and diversity (B).

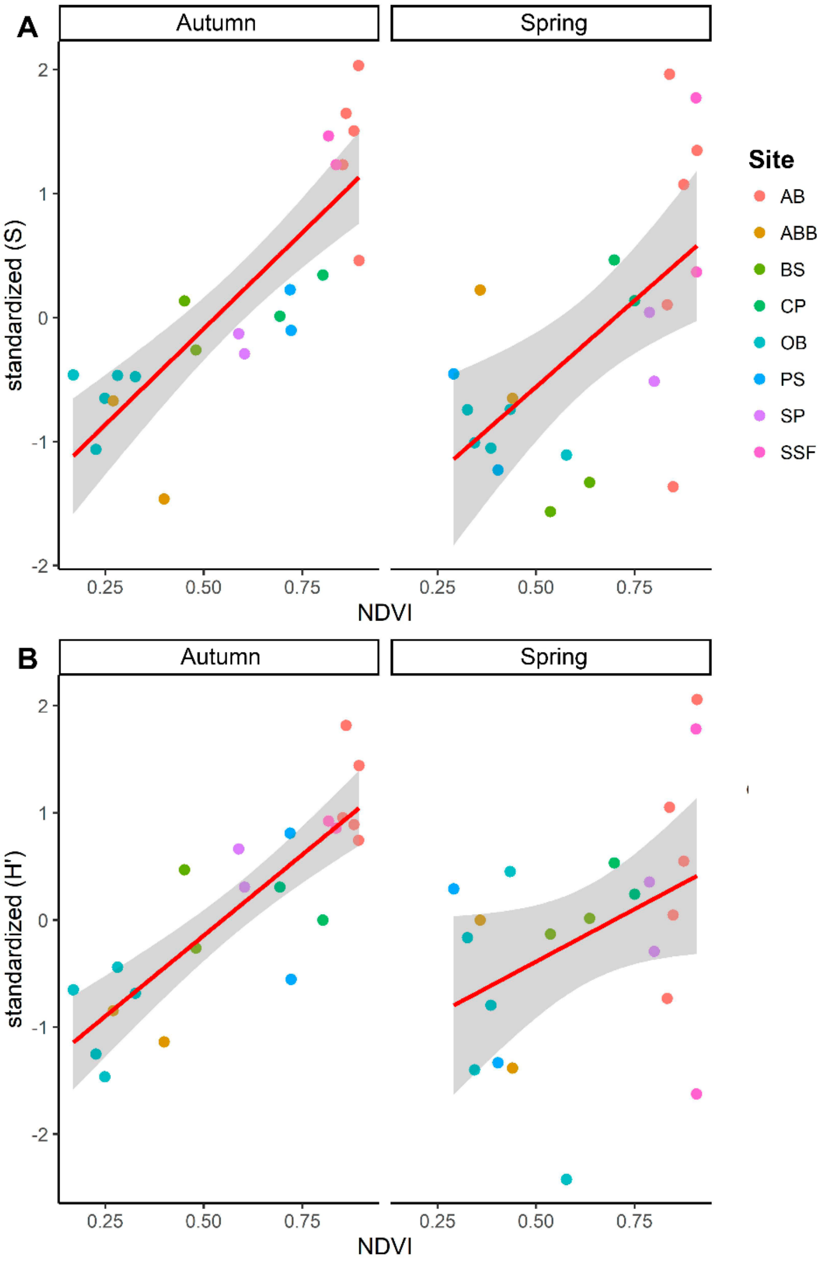

Supplement: Supplementary file 1 [file jof-07-00587-s001.zip › jof-1304115 revision supplementary/revision supplementary.figure.pdf]
